# Insanity and Its Treatment

**Published:** 1886-06

**Authors:** 


**Insanity and Its Treatment.** By G. FIELDING BLANDFORD, M. D., Oxon. F. R. C. P., Lecturer on Psychological Medicine, St. George's Hospital, London. Third Edition, together with Types of Insanity. An illustrated Guide to the Physical Diagnosis of Mental Disease, by ALLEN MCLANE HAMILTON, M. D., one of the Consulting Physicians of the Insane Asylum of New York City. New York: Wm. Wood & Co. 1886.

This volume of Wood's library will be generally welcomed as one of the most convenient "handy-books" on the subject obtainable. Originally written for delivery to a medical class, the lectures have been so amplified and improved in the successive editions of the book that, while making no claim to be a complete treatise on insanity, it contains the essentials needed by the general practitioner; and, considering its size, condensation and practical character, it deserves high praise. The addition of Dr. McLane Hamilton's treatise on "Types of Insanity," together with the excellent plates belonging to that work, increases the value of the book.

